# Characterization of Rumen Microbiota of Two Sheep Breeds Supplemented With Direct-Fed Lactic Acid Bacteria

**DOI:** 10.3389/fvets.2020.570074

**Published:** 2021-01-15

**Authors:** Sinalo Mani, Olayinka A. Aiyegoro, Matthew A. Adeleke

**Affiliations:** ^1^Gastrointestinal Microbiology and Biotechnology Unit, Agricultural Research Council - Animal Production, Irene, South Africa; ^2^Discipline of Genetics, School of Life Sciences, College of Agriculture, Engineering and Science, University of KwaZulu-Natal, Durban, South Africa; ^3^Research Unit for Environmental Sciences and Management, North-West University, Potchefstroom, South Africa

**Keywords:** 16S rRNA gene sequencing, lactic acid bacteria, sheep breed, microbial community, rumen

## Abstract

Supplementation of direct-fed microbials into ruminants' nutrition has shown great potential in manipulating rumen fermentation and enhancing productive animal performance. However, little is known about rumen microbial composition and diversity of Damara and Meatmaster sheep, breeds indigenous to South Africa. The study aimed at exploring and comparing the rumen microbiomes of two breeds with different feeding treatments as follows: no antibiotic, no probiotics (T1), only potential probiotic (T2), only potential probiotic (T3), the combination of potential probiotics (T4), antibiotic (T5); using a metagenomic approach. The results showed that based on the Shannon index, the microbial diversity of Damara was higher (*p* < 0.05) than Meatmaster, while treatment T4 was higher than treatment T1 (*p* < 0.05). The principal coordinate analysis showed no significant difference among treatments, while there were significant dissimilarities between sheep breeds and sample-day (*p* < 0.05). Canonical correspondence analysis (CCA) displayed the dispersion of microbial communities among treatments, where negative control (T1) was distinct from other treatments. *Bacteroidetes* and *Firmicutes* were the most abundant microbial phyla across treatments for both breeds. Negative control and the combination of potential probiotics showed lower proportions of *Proteobacteria* compared to other treatments. At the genus level, *Prevotella* and *Clostridium* were abundant across all treatments, while *Pseudomonas* was abundant only in T2, T3, and T5. In all treatments, *Fibrobacter* was detected after the feeding trials, while it was not detected in most treatments before trials. The results revealed that the rumen microbiome's structure and abundance were slightly altered by administering lactic acid as a putative probiotic.

## Introduction

The rumen is an important digestive and metabolic site in ruminants, where rumen microbiota play a vital role in providing nutrients to the host in form of volatile acids and microbial proteins (1). The symbiotic microbial community is essential for the host health in several ways, such as in nutrient digestion, balancing the immune response, and mediating the host functioning. Furthermore, symbiosis enables the development of the latter's gastrointestinal tract (2). Consequently, alterations in ruminal microbial structure and function have shown an insightful impact on ruminants' health and productivity. Although rumen microbiota is relatively stable, it is greatly responsive to changes in the diet, host genetics, physiology, and environmental factors (3, 4).

Feed additives such as direct-fed microbials have shown great potential to manipulate rumen fermentation and enhance productive animal performance (5). Antibiotics as growth promoters in animals introduce residues and spread antibiotic resistance while enhancing growth performance (6). Antibiotic administration disrupts the ruminant's normal microbial balance, which might cause growth defects in animals (7). Probiotics are explored as safer alternatives to antibiotics to improve balance in the gastrointestinal microbiota and enhance ruminants' health and productivity (8). Previous studies have shown that the use of probiotics (direct-fed microbials), such as lactic acid bacteria, can improve nutrient digestibility and growth performance (9, 10); also decrease pathogen colonization in the gut (11).

Earlier studies reported that probiotics enhanced feed efficiency, increased weight gain, improved milk and meat production, reduced methane emission, and animal health (5, 12–15). However, limited studies have explored the rumen microbial community composition and diversity. A study by Zhang et al. (16) showed that *Lactobacillus rhamnosus* GG administration to neonatal calves increased their voluntary feed intake and growth performance. The ruminal pH of calves fed the probiotic was lower, which might be caused by increased volatile fatty acids (VFA) concentration in rumen fluids. The *L. rhamnosus* GG administration also varied the rumen microbial community composition and regulated the balance of rumen and intestinal microbes. The relative abundance and order of dominant bacteria families were inconsistent between the control and probiotic calves. *Prevotellaceae* (55.67%) had the highest relative abundance in the treated group of calves instead of *Succinivibrionaceae*, and it significantly increased while *Succinivibrionaceae* decreased drastically. The relative abundance of *Lactobacillaceae* increased in the rumen fluid after the GG administration in calves.

In recent years, high-throughput sequencing of the 16S rRNA gene has provided novel insights into the abundance and diversity of microbial communities. It has also expanded the possibilities for studying rumen microbial community and population dynamics, indicating their significant role in ruminal fermentation (17, 18). Knowledge of these microbiomes can further broaden our understanding of rumen microbial environments and ruminant productivity. Hence the current study aimed at exploring the rumen microbial diversity and abundance by describing and comparing the rumen microbiota of sheep breeds subjected to different feed supplements with lactic acid bacteria as direct-fed microbials.

## Materials and Methods

### Animals, Treatments, and Sampling

All the procedures involving animals were approved by the Agricultural Research Council - Animal Production Institute Ethics committee (APIEC17/21). The trials were done at the Agricultural Research Council (ARC), GI Microbiology and Biotechnology unit and the Small Stocks Unit in Irene, Gauteng province.

The lactic acid bacteria (putative probiotics) used in this study were isolated and characterized from fresh fecal samples of six Zulu sheep breed. These sheep have been described to possess great adaptation to harsh environmental conditions and resistance against parasites and diseases. Molecular sequencing and probiotic and technological properties such as antimicrobial activity, acid, and bile tolerance were used to identify the two potential probiotics. The potential probiotic bacteria were prepared in De Man, Rogosa, and Sharpe (MRS) broth (Oxoid, England) anaerobically and preserved in 25% glycerol in the ultra-low freezer. The two potential probiotic bacteria were then revived by inoculation in MRS broth. For suspension, MRS broth was inoculated with 1% (v/v) culture and incubated anaerobically at 37°C overnight prior to administering.

Sixty-four sheep, 32 Damara breed, and 32 Meatmaster breed were used in a 30-day trial. The animals were approximately 7 months old, with 16 males and 16 females per breed. Ten (10) animals were excluded from the experiment after the adaptation period before the trial commenced due to signs of sickness and four mortalities. The average initial weights of the animals were: Meatmaster males (24.6 ± 3.4 kg), Meatmaster females (21.5 ± 3.1 kg), Damara males (36.6 ± 8.3 kg), and Damara females (28.9 ± 6.9 kg). The animals were housed per treatment with males and females separated in open barn trial pens with ± 4 m^2^ shelters.

The sheep were randomly allocated to five treatment groups, 6–8 animals per treatment, considering the gender. The treatments were as follows: (a) Diet with no antibiotics, no probiotics (negative control) (T1); (b) Diet with no antibiotics, only *L. rhamnosus* PT9 (T2); (c) Diet with no antibiotics, only *L. rhamnosus* PT10 (T3); (d) Diet with the combination of *L. rhamnosus* PT9 and *L. rhamnosus* PT10 (T4); (e) Diet with antibiotics, no probiotics (positive control) (T5). The animals were fed on commercial pellet feed fortified with or without probiotics or antibiotics, and in case of the positive control, in-feed antibiotic rumensin was added. Hay and freshwater were supplied *ad libitum*. The experimental feed composition is presented in Table 1, feed composition is analyzed as follows: Dry matter (19), Moisture (19), Protein (19), NDF (20), ADF and NDL (21), Ash (19), Starch (22), Crude Fiber (19), Calcium and Phosphorus (23). The probiotic treatment groups were dosed once a week for the trial period using the dosing gun with 10 mL of the 24-h old LAB culture suspensions of approximately 2 × 10^9^ CFU/mL. Animal weight was recorded, and rumen samples were collected before the trial commenced and at the end of the trial.

**Table 1:** Ingredient and chemical composition of experimental diet fed to sheep.

**Nutrients**	**Composition (g/kg)**	**Ingredient composition (g/kg DM)**
Dry matter	893.3	Corn silage 600, Corn grain finely ground 250, Soybean meal 150, Urea:ammonium sulfate (9:1) 6.1
Moisture	106.8	
^*^Protein (*N* × 6.25)	152.8	
Fat (ether extraction)	15.2	
NDF (neutral detergent fiber)	370.9	
ADF (acid detergent fiber)	134.9	
ADL (acid detergent lignin)	55.9	
Ash	89.2	
Starch	327.1	
Fiber (crude)	123.8	
Calcium	13.9	
Phosphorus	3.20	

* *For the conversion of nitrogen to protein content the factor 6.25 was used*.

Rumen fluid samples were collected using a stomach tube, according to Shen et al. (24) procedures. About 40 mL of the collected rumen fluid contents were transferred to 50 mL centrifuge tubes after collection. The rumen pH was measured using a portable pH meter (Orion Star A121 Portable pH meter, Thermo Scientific, Singapore) immediately after sampling (before and after the trial). The pH meter was calibrated using fresh pH buffers that bracket expected sample pH using automatic buffer recognition according to manufacturer's guidelines. Before each sample reading, the pH meter was rinsed with distilled water and blot dried, rinsed again after each sample reading. Rumen samples were kept on ice until they were transferred to the lab, and they were stored at −80°C until further analysis.

### DNA Extraction and 16S rRNA Amplification

Extraction of DNA from rumen samples (100 μl each) was done using QIAamp Fast DNA stool Mini Kit (Qiagen, Germany) according to the manufacturer's guidelines for pathogen detection, and DNA concentration was evaluated with Nanodrop 2000 (Thermo Electron Corporation, USA). The DNA samples were pooled according to their respective treatments, considering the sex of the animals (males and females DNA pooled separately), leading to four samples per treatment (two females and two males), and 20 samples per breed. The pooled DNA quality was assessed by electrophoresis on 1.5% agarose gels and visualized with UVP BioSpectrum 310 Imaging System (FisherScientific, UK). The pooled DNA samples were used as templates for amplifying a partial 16S rRNA sequence using the following primers, which include Illumina overhang adapter sequences:

Forward = 5'TCGTCGGCAGCGTCAGATGTGTATAAGAGACAGCCTACGGGNGGCWGCAG3', Reverse = 5'GTCTCGTGGGCTCGGAGATGTGTATAAGAGACAGGACTACHVGGGTATCTAATCC3' targeting the highly variable V3–V4 region of the prokaryotic 16S rRNA gene. PCR was done in 25 μl reaction mixture comprising 5 μl template DNA, 1 μl of each primer, 5.5 μl nuclease-free water, and 12.5 μl of 2 × KAPA HiFi HotStart Ready Mix (KAPA Biosystems), with the following conditions: initial denaturation at 95°C for 3 min, 30 cycles of amplification at 95°C for 30 s, 55°C for 30 s, and 72°C for 30 s and a final extension at 72°C for 5 min. PCR amplicons were visualized using agarose gel electrophoresis to verify the expected band size of 550 bp. PCR products were purified to remove any primer dimers with NucleoSpin Gel and PCR Clean-up kit (Macherey-Nagel, Germany).

### Library Preparation and Sequencing

Sequencing libraries were generated, and index codes were added using Illumina MiSeq Nextera XT DNA Library Preparation Kit (Illumina, USA), according to manufacturer's guidelines. The library quality was evaluated using Qubit 2.0 Fluorometer (ThermoScientific, USA) and Agilent 2100 Bioanalyzer system. Sequencing was performed using Illumina MiSeq platform, and 300 bp paired-end reads were generated.

### Data Analysis

The raw data sequences generated by MiSeq Illumina sequencer were trimmed using Trimmomatics version 0.36, where the low-quality sequence regions and Illumina universal adapter sequences were removed. Demultiplexed sequence files were imported to QIIME2 software (version 2018.8) (25) for analysis. The imported reads were denoised and trimmed using DADA2. Sequences with ≥97% similarity were assigned to the same operational taxonomic units (OTU), and the OTU feature table was generated; chimeric sequences were detected and removed from the representative OTU sequences. For taxonomic analysis, OTU representative sequences were aligned to the Greengenes database. All other statistical analyses were carried out using RStudio (version 3.5.3) with phyloseq package (version 1.24.2). OTUs at a relative abundance ≥ 0.05% of the total reads in at least one sample were retained and analyzed further in RStudio.

Alpha diversity metrics including species richness, Simpson and Shannon index were computed for all samples. Beta diversity was computed using distance matrices generated from unweighted UniFrac analysis, principal coordinates analysis (PCoA), and non-metric multidimensional scaling (NMDS). ANOVA was used to compare the univariate analysis of alpha diversity measures. The probability of error α was fixed at 5%.

A co-occurrence network was constructed to determine potential associations between microbial communities. A pairwise correlation was calculated between microbial abundances present in at least 25% of the samples and with abundance higher than 0.05%, using Spearman's co-efficient, using Pairwise_correlations.R and Network_Analysis.R scripts (26). Multiple testing correction was done to reduce the chances of obtaining false-positive results; the *P*-values were adjusted using the Benjamini–Hochberg standard false discovery rate correction (FDR-BH) method (27). Spearman's coefficient correlations (ρ) ≥ 0.7 (or ≤ -0.7), and *p*-value < 0.01 were considered significant. The interactive platform Gephi (28) was used to visualize the network structure, using undirected network and Fruchterman–Reingold layout. Modularity was calculated by the Louvain method (29), using the clustering algorithm implemented in Gephi; modules were then filtered to remove nodes with a degree < 2. Betweenness centrality was used to measure the centrality of each node in the network.

Sequences for the two potential probiotics have been deposited on NCBI; *L. rhamnosus* PT9 and *L. rhamnosus* PT10 accession numbers MT492059 and MT492060, respectively. Raw reads used in the present study were deposited to the National Center of Biotechnology Information (NCBI) Sequence Read Archive (SRA) database under BioProject PRJNA578022 (https://www.ncbi.nlm.nih.gov/bioproject/?term=PRJNA578022).

## Results

Our results revealed that treatment, breed, and their interactions affected body weight (*p* < 0.001) and pH (*p* < 0.05) of the sheep; only breed was significant (*p* < 0.005) on weight gain. In Table 2, the final bodyweight of the control group (T1) was lower (*p* < 0.05) than other treatment groups. On average, bodyweight in other treatment groups was higher, with about 4.85 kg relative to the control group. Ruminal pH was higher (*p* < 0.05) in T5 than the probiotic-supplemented groups, but statistically the same with the control group. Except for T2, animals in T3, T4, and T5 groups gained more weight, with the highest weight gained being 1.7 kg in T4 within the 30-day trial. Damara breed was 12 kg heavier, 1.3 kg higher, and 0.34 higher than Meatmaster for body weight, weight gain, and pH, respectively, as shown in Table 3.

**Table 2:** Effect of treatment on body weight, weight gain and ruminal pH of sheep.

**Parameter**	**Treatment**				
	**T1**	**T2**	**T3**	**T4**	**T5**
*n*	8	9	9	14	14
Initial body weight (kg)	24.0 ± 6.2	28.2 ± 9.6	29.7 ± 9.1	28.4 ± 10.3	28.3 ± 5.5
Final body weight (kg)	26.5^b^ ± 7.6	30.4^a^ ± 9.7	32.5^a^ ± 9.6	32.6^a^ ± 10.7	31.2^a^ ± 5.8
Weight gain (kg)	2.5^b^ ± 2.2	2.2^b^ ± 2.1	2.8^ab^ ± 1.8	4.2^a^ ± 1.6	2.9^ab^ ±1.3
Initial ruminal pH	7.13 ± 0.16	7.12 ± 0.27	7.12 ± 0.3	7.07 ± 0.4	7.31 ± 0.3
Final ruminal pH	7.19^ab^ ± 0.14	7.11^b^ ± 0.2	7.03^b^ ± 0.24	7.15^b^ ± 0.3	7.23^a^ ± 0.2

*Treatments: T1- diet with no probiotic, no antibiotic (negative control); T2-diet with Lactobacillus rhamnosus PT9; T3- diet with Lactobacillus rhamnosus PT10; T4- diet with combination of Lactobacillus rhamnosus PT9 and Lactobacillus rhamnosus PT10; T5- diet with antibiotic (positive control)*.

a, b *Mean values in a row with different subscripts are significantly different p < 0.05. n: number of observations, ±SD- standard deviation*.

**Table 3:** Effect of breed on body weight, weight gain and ruminal pH of sheep.

**Parameter**	**Breed**	
	**Damara**	**Meatmaster**
*n*	22	32
Initial Body weight (kg)	35.0 ± 7.9	23.0 ± 3.5
Final Body weight (kg)	38.8^a^ ± 7.9	25.5^b^ ± 4.1
Weight gain (kg)	3.8^a^ ± 1.5	2.5^b^ ± 1.9
Initial ruminal pH	7.38 ± 0.29	7.0 ± 0.21
Final ruminal pH	7.35^a^ ± 0.15	7.01^b^ ± 0.18

*n: number of observations*.

a, b *Mean values in a row with different subscripts are significantly different *p* < 0.05, ±SD- standard deviation*.

**Figure 1:**
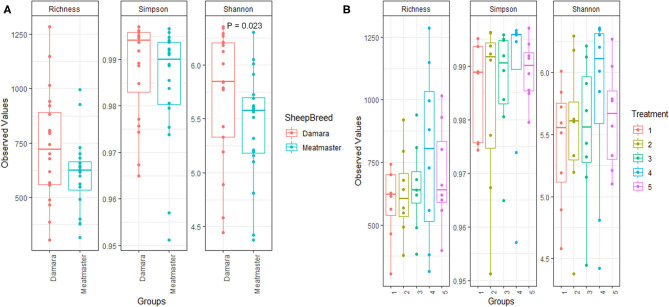
Comparison of alpha diversity metrics of communities by microbial richness, species diversity (Simpson and Shannon indexes). Treatments groups are as follows: 1. Diet with no antibiotics, no probiotics (negative control); 2. Diet with no antibiotics, only *Lactobacillus rhamnosus* PT9; 3. Diet with no antibiotics, only *Lactobacillus rhamnosus* PT10; 4. Diet with the combination of *Lactobacillus rhamnosus* PT9 and *Lactobacillus rhamnosus* PT10; 5. Diet with antibiotics, no probiotics (positive control). **(A)** Comparison of within community, grouped by sheep breed. **(B)** Comparison of within community, grouped by treatment. Shannon index was significant only between treatment 4 and treatment one, *P* = 0.042.

After quality control, a total of 1,996,134 reads were obtained in 40 samples, with an average of 49,903 reads per sample. A total of 11 202 OTUs were obtained, out of which only 9,244 OTUs were classified at phylum level and were used for further analysis. The rest of 1,958 OTUs could be classified only up to kingdom level and they were discarded after normalization. The distinct variation by the two Damara samples was observed in the rarefaction curve (Figure 1), indicating a difference in of Damara and Meatmaster sheep breeds' microbial composition.

**Figure 2:**
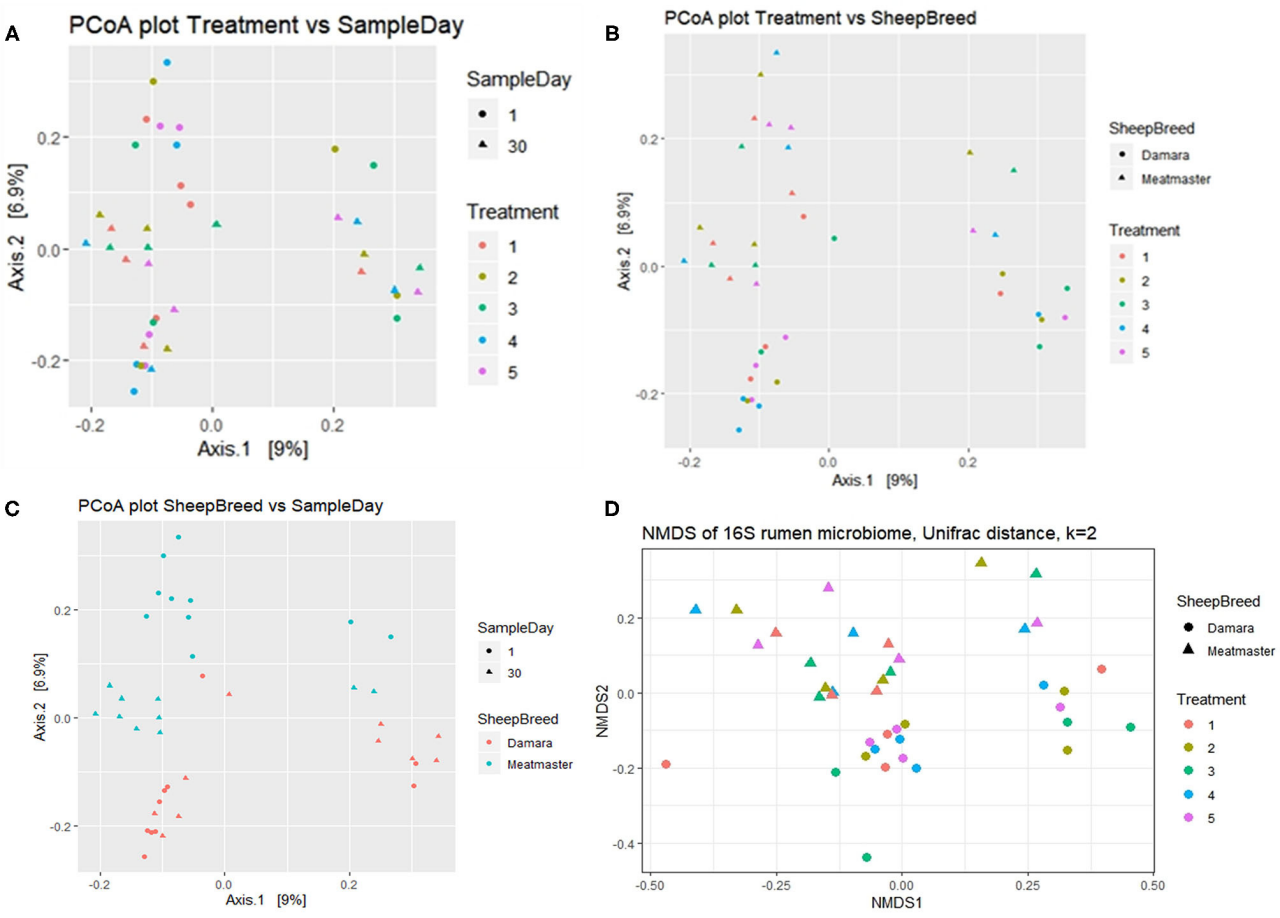
Beta diversity of rumen microbiome of Damara and Meatmaster sheep breeds. Treatment groups are as follows: 1. Diet with no antibiotics, no probiotics (negative control); 2. Diet with no antibiotics, only *Lactobacillus rhamnosus* PT9; 3. Diet with no antibiotics, only *Lactobacillus rhamnosus* PT10; 4. Diet with the combination of *Lactobacillus rhamnosus* PT9 and *Lactobacillus rhamnosus* PT10; 5. Diet with antibiotics, no probiotics (positive control). **(A)** Principal coordinate analysis (PCoA) defining the relationship between samples based on treatment and sample day. **(B)** PCoA defining the relationship between samples based on treatment and breed. **(C)** PCoA defining the relationship between samples based on sample day and breed. **(D)** Non-metric multidimensional scaling (NMDS) analysis based on the Bray-Curtis similarity index.

The alpha diversity (Richness, Simpson and Shannon indexes) is presented in Figure 1, showing indices across breed and treatment, respectively. Damara sheep had higher (*p* < 0.05) Shannon index than Meatmaster sheep (Figure 1A); while other indices showed no significant difference between the breeds, the microbial richness in Damara sheep was higher than in Meatmaster sheep. The combination of potential probiotics increased the microbial richness and diversity as treatment 4 had higher Shannon index than treatment 1 (*p* < 0.05, Figure 2B).

**Figure 3:**
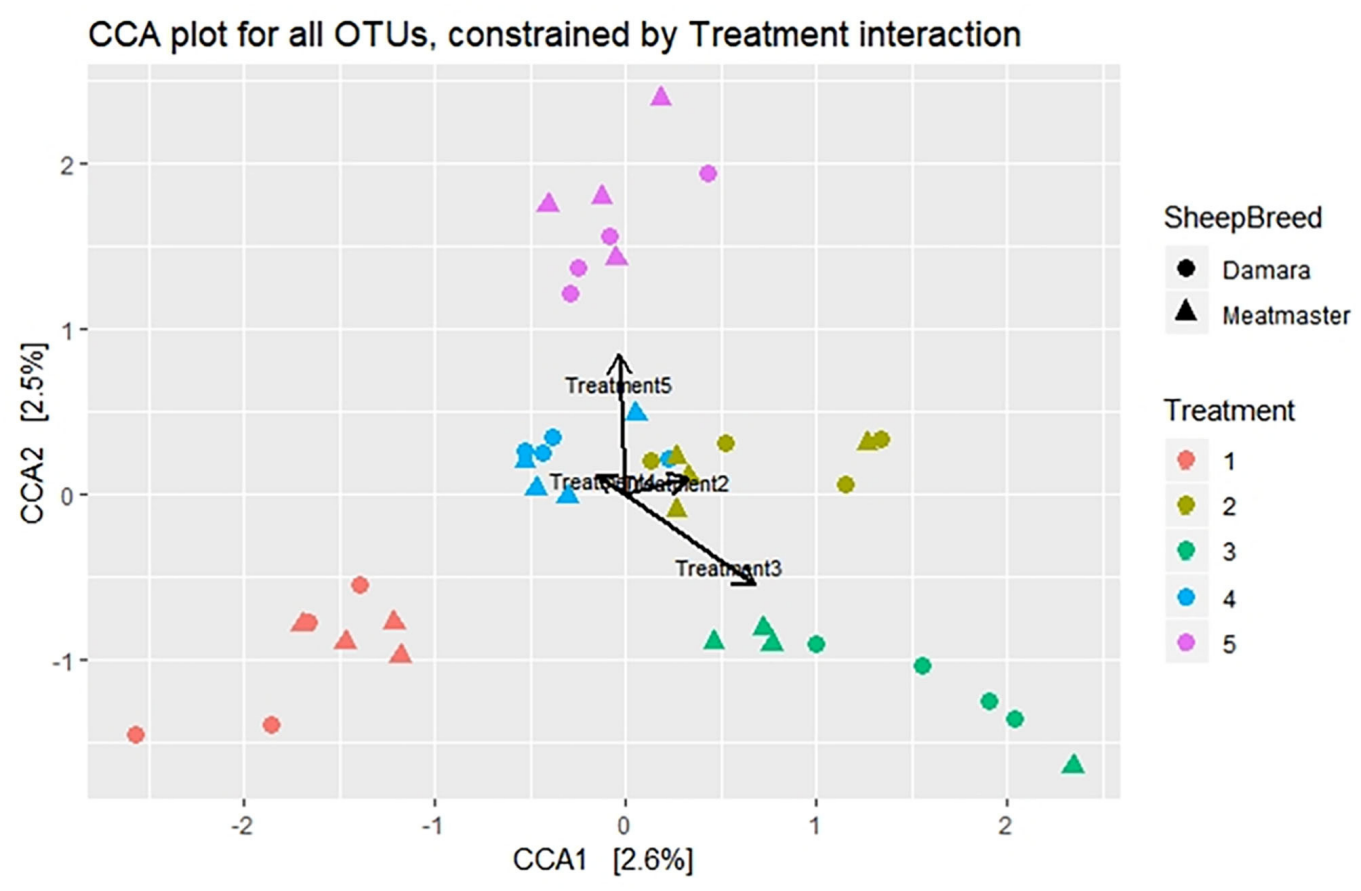
Canonical correspondence analysis (CCA) visualizing the correlation of the microbiome structures between treatments. Treatment groups are as follows: 1. Diet with no antibiotics, no probiotics (negative control); 2. Diet with no antibiotics, only *Lactobacillus rhamnosus* PT9; 3. Diet with no antibiotics, only *Lactobacillus rhamnosus* PT10; 4. Diet with the combination of *Lactobacillus rhamnosus* PT9 and *Lactobacillus rhamnosus* PT10; 5. Diet with antibiotics, no probiotics (positive control). Arrows show the relationship between treatments.

Beta diversity was calculated using the UniFrac dissimilarity and presented by PCoA (Figures 2A–C) and NMDS (Figure 2D). There were no dissimilarities (*p* > 0.05) detected in the rumen microbial community between the treatment groups and no clustering pattern was demonstrated between the treatments. Instead, dissimilarities were detectable between the sheep breeds (*p* < 0.05) and sample day (*p* < 0.05). The samples were clustered according to sample day or breed; few Meatmaster samples exhibited slightly higher diversity than other animals. The CCA (Figure 3) was used to identify the association between the treatment groups. Though the dissimilarities between the treatment groups were not significant, CCA showed that the microbial community from T1 was distinct from the microbial communities of other groups. The microbial communities for T2 and T4 were overlapping.

**Figure 4:**
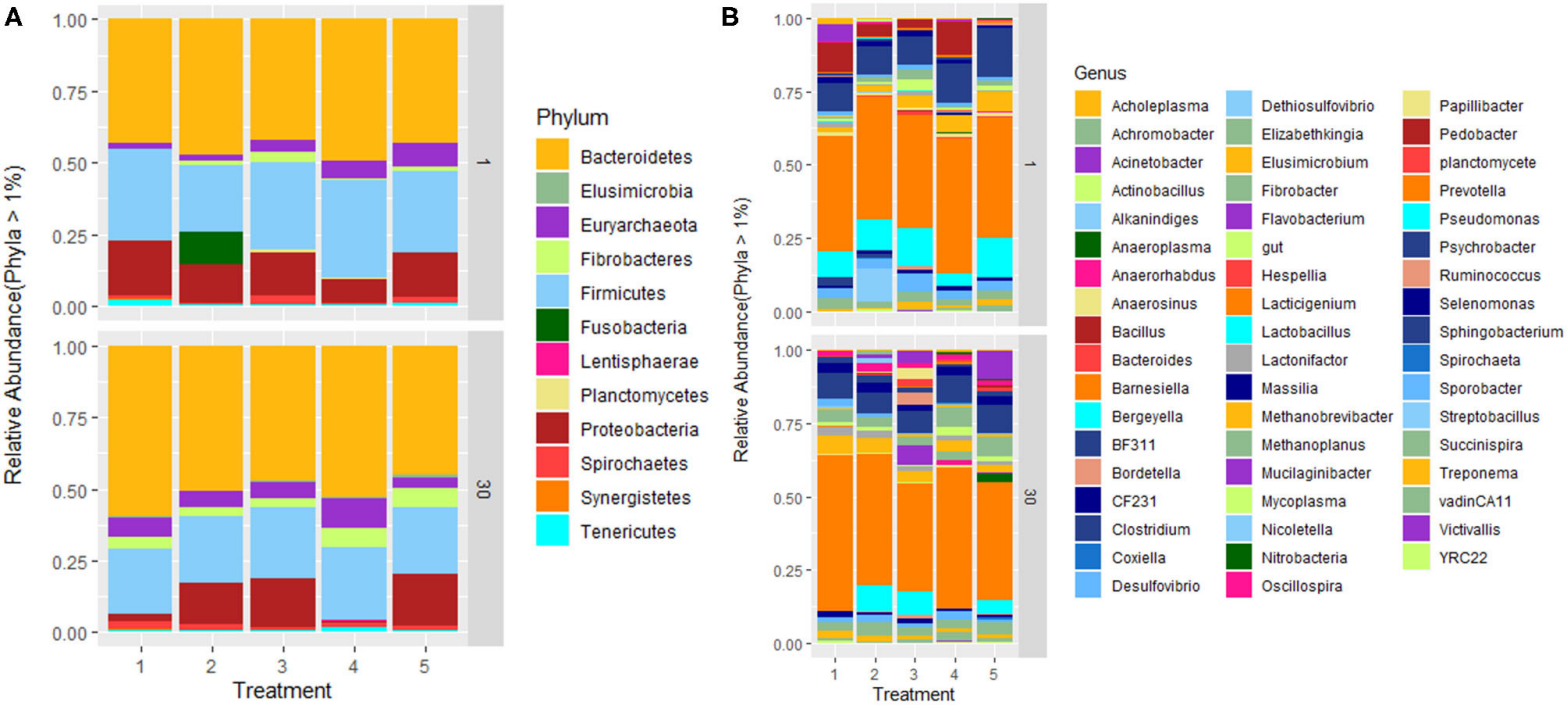
Relative abundance of rumen microbial communities at **(A)** phylum and **(B)** genus level. Treatment groups are as follows: 1. Diet with no antibiotics, no probiotics (negative control); 2. Diet with no antibiotics, only *Lactobacillus rhamnosus* PT9; 3. Diet with no antibiotics, only *Lactobacillus rhamnosus* PT10; 4. Diet with the combination of *Lactobacillus rhamnosus* PT9 and *Lactobacillus rhamnosus* PT10; 5. Diet with antibiotics, no probiotics (positive control).

The abundance of microbiota and its taxonomic distribution was estimated in all treatments. Fifteen (15) bacterial phyla and 1 archaeal phylum were detected across all samples at a relative abundance of ≥0.05%. At phylum level; after the trials, *Bacteroidetes* were the highest abundant phenotype across all 5 treatment groups (1–5) accounted for 51%; 46.3%; 41.9%; 46.2% and 43.95% of relative abundance, respectively, followed by *Firmicutes* that accounted for 30.5%; 29.9%; 28.6%; 31.7% and 25.5% of relative abundance; and *Proteobacteria* that accounted for 4.77%; 13.83%; 19.82%; 4.92% and 17.41% of relative abundance. Treatments 1 and 4 had lower numbers of *Proteobacteria* compared to other treatments. *Euryarchaeota* abundance accounted for 2.31%; 1.7%; 1.78%; 3.02% and 1.82% respectively (Figure 4A). The supplementation of the combination of probiotics increased the percentage of Lentisphaerae (2.24%) in the rumen. At genus level, a total of 102 genera were identified in the rumen (Figure 4B). *Prevotella* and *Clostridium* were abundant across all treatment groups, while *Pseudomonas* was abundant only in treatments 2, 3, and 5. The relative *Fibrobacter* was detected in all treatment groups (3.98%, 3.47%, 5.74%, 5.97% and 6.33%, respectively) after the feeding trials, whereas it was detected in few treatment groups before the trials. Microbial relative abundance was diverse across treatments.

**Figure 5:**
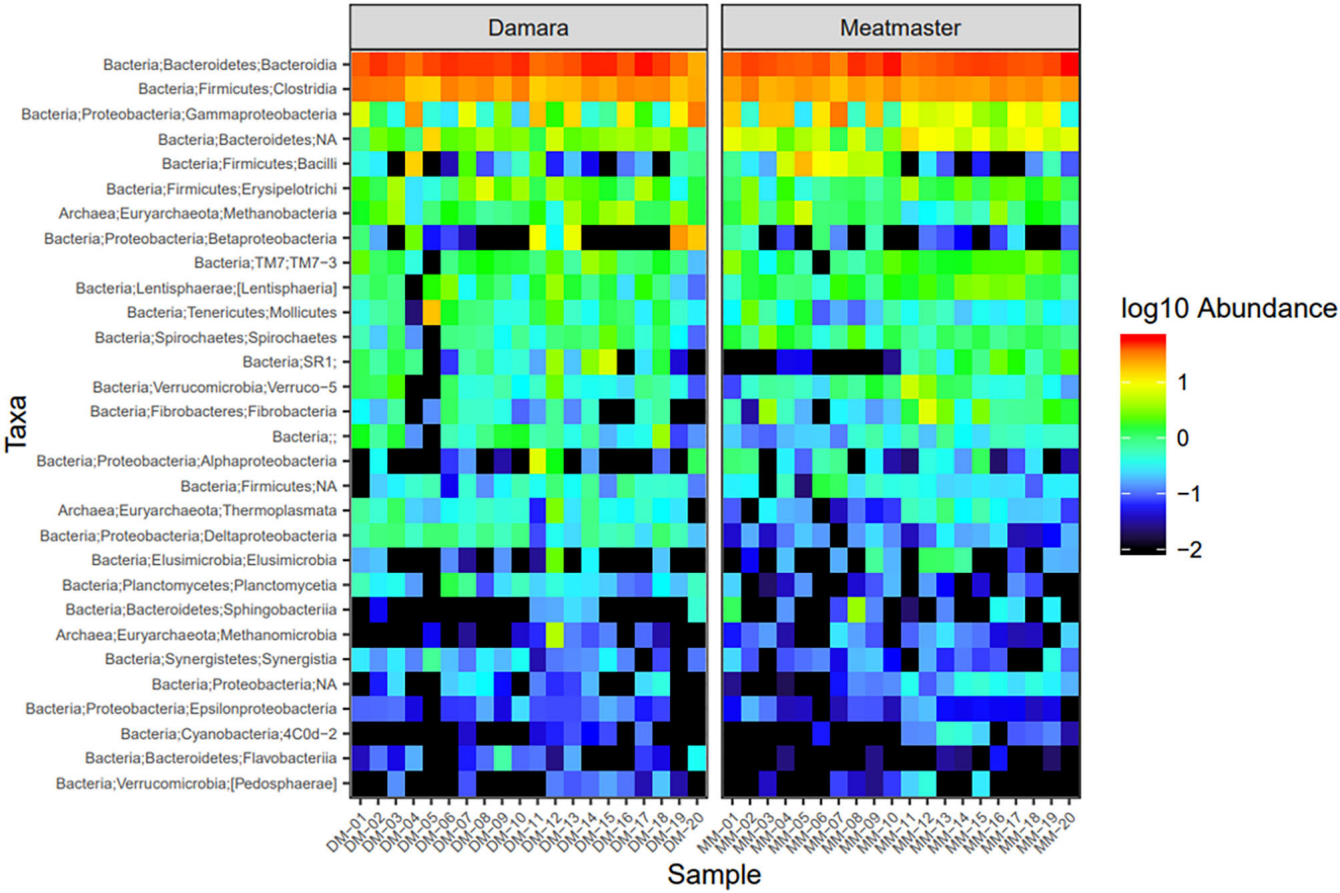
Heatmap of the top 30 most abundant taxa by class between the two sheep breeds. DM1-DM10 and MM1-MM10 are samples before the treatments were administered, DM11-DM20 and MM11-MM20 are samples after the trial. Relative values for each genus depicted by color intensity according to the legend provided on the scale of −2 to 1.

**Figure 6:**
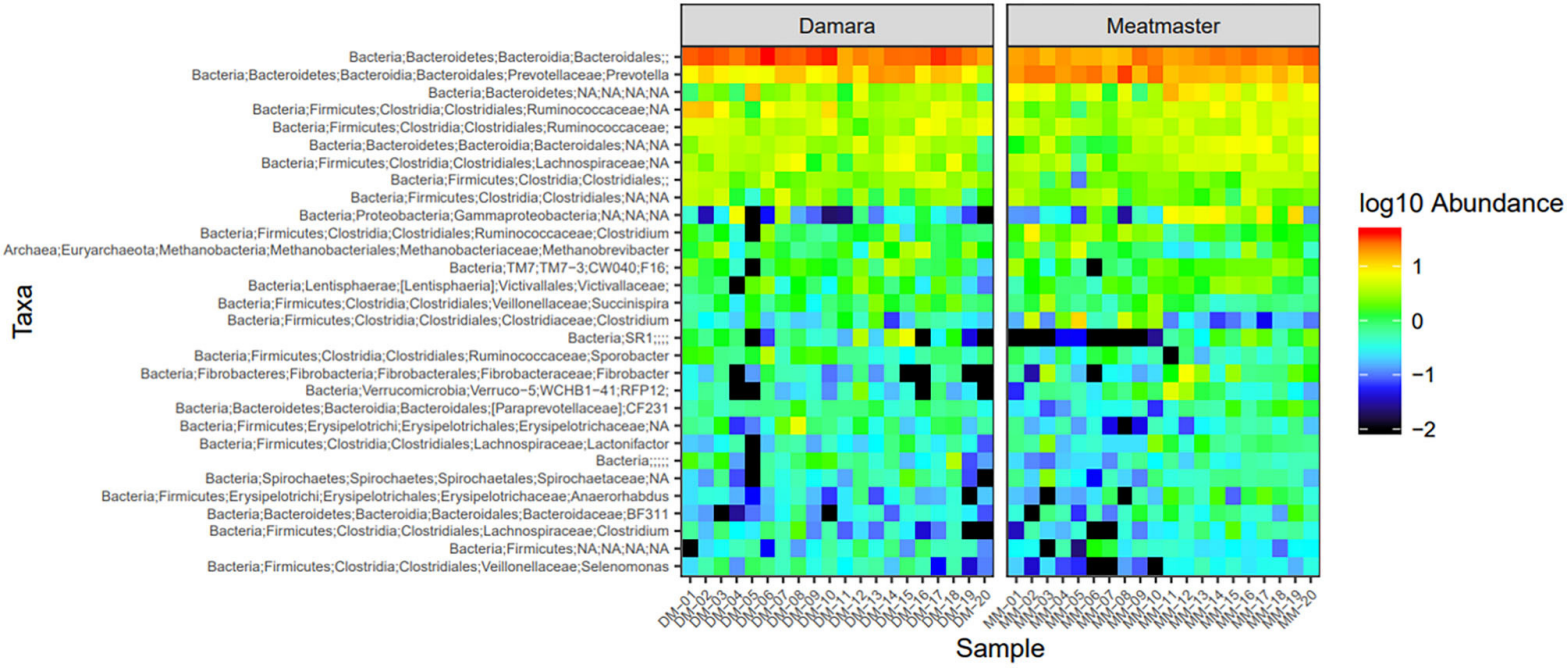
Heatmap of the top 30 most abundant taxa by genus between the two sheep breeds. DM1-DM10 and MM1-MM10 are samples before the treatments were administered, DM11-DM20 and MM11-MM20 are samples after the trial. Relative values for each genus depicted by color intensity according to the legend provided on the scale of −2 to 1.

**Figure 7:**
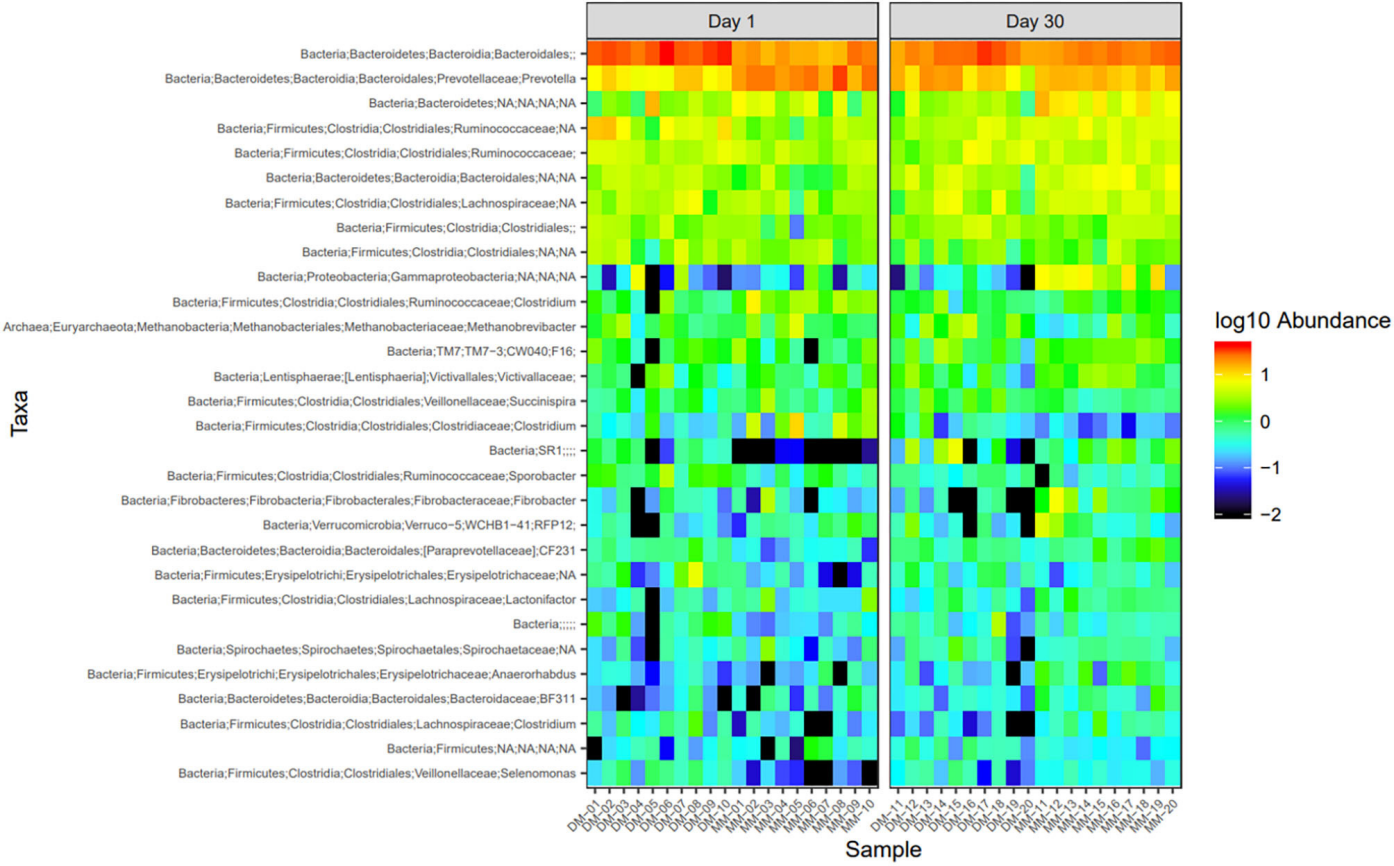
Heatmap of the top 30 most abundant taxa by genus between sampling days. DM1-DM10 and MM1-MM10 are samples before the treatments were administered, DM11-DM20 and MM11-MM20 are samples after the trial. Relative values for each genus depicted by color intensity according to the legend provided on the scale of −2 to 1.

Microbial abundance was further evaluated using heatmap analysis for top 30 microbial community at class (Figure 5) and genus (Figure 6) levels for Damara and Meatmaster sheep breeds as well as for sample period (Figure 7). At class level, a total of 32 classes were identified. *Bacteroidia* and *Clostridia* were most abundant in both breeds. Gammaproteobacteria accounted for 7.3% in Damara and 8.9% in Meatmaster sheep. TM7-3 remained unchanged in Damara sheep, and it increased in Meatmaster from 1.4 to 2.35% after the trials. Fibrobacteria and Verruco-5 were steady in Damara sheep, while they increased in Meatmaster (0.6–2.17% and 0.78–1.49%, respectively). Bacilli decreased in both breeds after the 30-day trial; 2.04–0.76% in Damara and 5.33–0.2% in Meatmaster.

At genus level, *Bacteroidales* and *Prevotella* were the most abundant taxa across the two breeds. Pseudomonas accounted for 15.1% in Meatmaster, while Damara had 5.8%. *Proteobacteria* phylum was more abundant in Meatmaster as compared to Damara sheep. This phylum consists of most pathogenic bacteria and Damara sheep are known to be resistant to parasites, which could be the reason for low quantities of *Proteobacteria* in Damara sheep.

**Figure 8:**
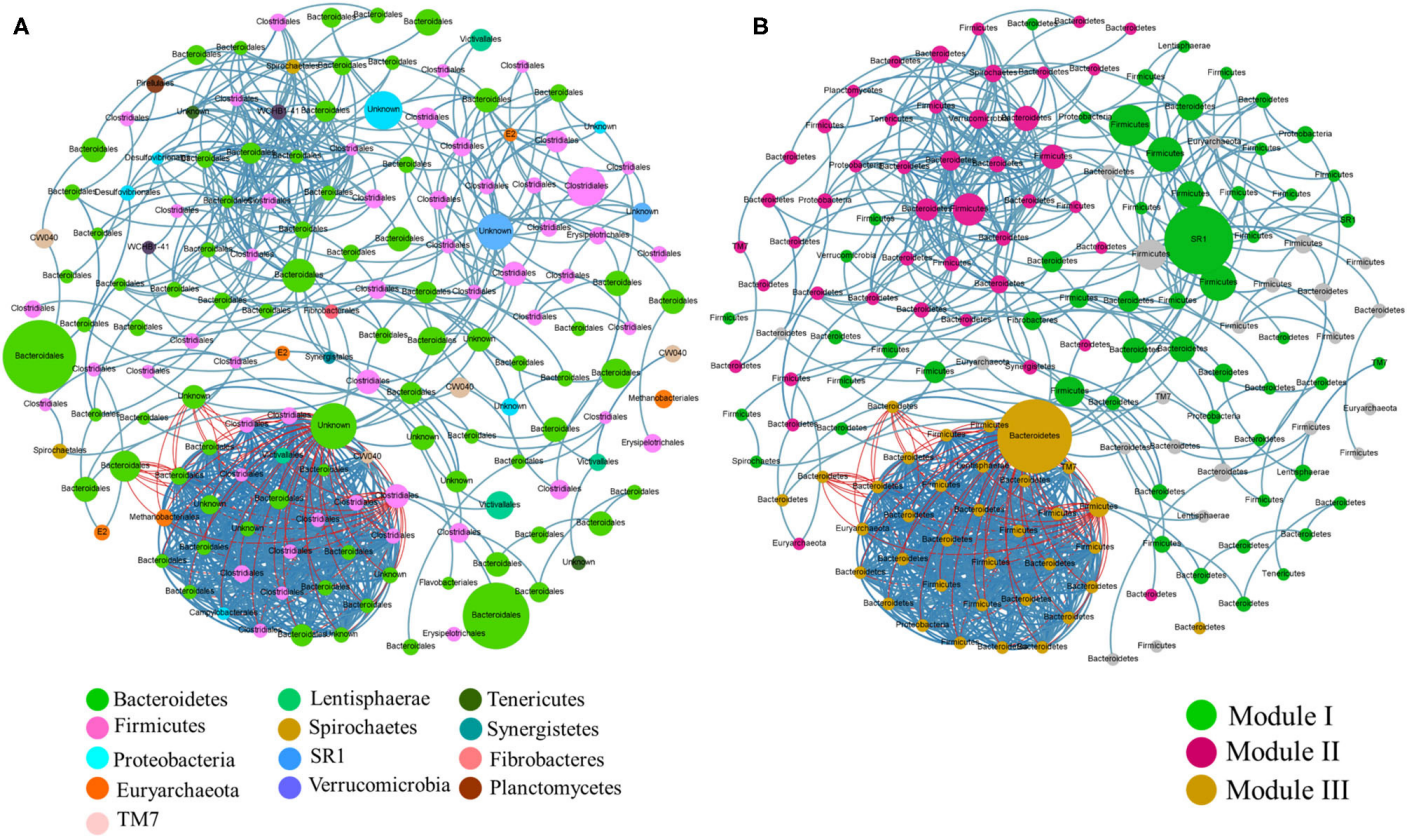
A Network analysis of rumen microbial communities. A connection stands for Spearman correlation with a value above 0.7 between microbial abundances. Edge color represents positive (blue) and negative (red) correlations and the edge thickness is equivalent to the correlation values. **(A)** Node color corresponds to the phylum taxonomic classification and the size of the node is proportional to the abundance of the order. **(B)** Node color corresponds to the modularity class and the node size is proportional to betweenness centrality.

A co-occurrence network was observed for microbial communities using Spearman correlation ( ≤ -0.7 or ≥0.7 and *p*-value of 0.01). The network had 158 nodes with a significant number of positive correlations compared to negative correlations. Each node represented a taxon with the node size comparative to its relative abundance in the sample (Figure 8A). The network displayed a modular structure with modularity of 0.532. A module depicts a microbial taxa subnetwork that potentially interacts or shares the same ecological niche without direct interaction. Three major modules comprising correlated microbial taxa centered by unclassified SR1, *Clostridiales* and unclassified *Bacteroidetes* were observed (Figure 8B). These microbial taxa were identified based on their betweenness centrality within the network. Module III being the only module showing negative correlations with about 78 pairs. *Firmicutes* were the most source taxa with members that showed a strong negative correlation with other taxa, mostly with *Bacteroidetes*.

## Discussion

In recent years, the relationship between rumen microbiota and their host has been shown to play a significant role in the host's function and health (30). Previous studies have shown that certain microorganisms as direct-fed microbials can have beneficial effects on the host, thus improving the health and production performance of dairy cows, sheep and goats (31–34).

The supplementation of probiotics influenced the sheep's weight improvement as the negative control showed lower body weight than other treatment groups; T3 and T4 recorded the heaviest bodyweight, which implies that the probiotics may impact the digestion of nutrients. Treatment with the combination of probiotics (T4) showed greater weight gain than the control and T2. The observed comparative increase of body weight gain on microbial treated groups in this study cannot be explained by excess dry matter intake, as all experimental animals were given the same type and amount of feed. Instead, the body weight gain could be associated with nutrient digestion's effectiveness stimulated by probiotics with microbiota interactions. A study by Roodposhti AND Dabiri (35) reported that average weight gain was significantly greater for the synbiotic, prebiotic and probiotic treatments than the control. They observed that synbiotic-fed calves had greater weight gain than other treatments and no significant difference between prebiotic and probiotic treatments. Damara breed had heavier body weight gain than Meatmaster breed. The effect of breed on the sheep's body weight gain was also reported by Wilkes et al. (36), where they recorded a difference in weight gain of the breeds with the Damara gaining on average of 142 g/day and Merino at 80 g/day. However, no significant interaction was found between diet and breed, yet Damara sheep gained faster than Merino sheep in both diets.

Ruminal pH is the key guide signifying the internal balance in the rumen environment; hence it is vital to maintain a moderately stable pH to ensure efficiency in ruminal fermentation. In the present study, treatment and breed had a significant effect (*p* < 0.05) on ruminal pH. Damara breed recorded a higher ruminal pH value than Meatmaster breed; this might be because Damara can retain more fibrous constituents in the rumen for a longer period, permitting the cellulolytic microorganisms to degrade the feed's fibrous constituents thoroughly. Cellulolytic bacteria's ability to digest fibers is maintained by optimal ruminal conditions, where pH between 6 and 9 is best (37). The high ruminal pH recorded in the antibiotic-treated group (T5) in this study might be associated with a decrease of some lactic acid bacteria due to antibiotic supplementation, which may have increased the pH. Probiotics stabilized ruminal pH, which may have enriched microbial ecology and may have also led to increased nutrient absorption and lactate, resulting in improved weight gain. A study by Nocek et al. (38) reported that bacterial probiotics consisting of *Lactobacillus plantarum* and *Enterococcus faecium* prompted alterations in the ruminal pH of high grain-fed cows. Yet, the mechanism of the effects of the bacterial probiotics on rumen fermentation was unclear.

In the present study, we evaluated and compared rumen microbial abundance and diversity of five (5) treatment groups of Damara and Meatmaster sheep breeds. The results revealed that the structure and abundance of rumen microbial communities were altered by administering potential probiotics and antibiotics.

Alpha diversity results within treatments revealed that the combination of potential probiotics (T4) had higher microbial diversity than other treatments. This might be because other treatments were also treated with feed additives, even though they were not as effective as the synergetic effect of T4. Wang et al. (39) reported that the increased nutrient levels in the diet affected microbial richness, while no significant differences were detected in the diversity across the treatments. Jia et al. (40) showed no significant difference in microbial richness and diversity among the five treatment groups. Breed variation affected the alpha diversity also, as Damara had higher microbial richness and diversity than Meatmaster. These findings show that the host genotype does have a significant role in regulating the rumen microbial community structure. The results are in accordance with the reports from other ruminants that investigated rumen microbial diversity. Huang et al. (41) reported that rumen bacterial diversity in Tibetan sheep was greater than Gansu sheep based on the Shannon-Weiner and Simpson indexes. Another study also showed that water deer had the highest bacterial diversity than reindeer and goat (42).

Beta diversity showed no significant dissimilarities among the treatments, which could mean that there was no distinct diversity in the microbial communities of the treatment groups, whereas sample time and breed variation displayed significant diversity. These results agree with a study by Noel et al. (43), where they observed that only breed had a significant effect on methanogen community structure, while diet had no significant effect. A study by Xu et al. (44) observed a structural difference between before and after the probiotic intervention. Furthermore, the association among treatment groups was explored using CCA, which displayed that microbial community from the negative control (T1) was further distant from other treatments, while there was a close interaction among the probiotic treatment groups. This suggests that the probiotic used in treatment 2 was more effective in interacting with the rumen microbiota than the one from treatment 3, as the distance of treatment 2 with the combination group is lesser than treatment 3. Though the treatment groups were not diverse statistically, their interaction shows differences in the microbial communities and probiotic treated groups were more similar compared to the antibiotic-treated group and the negative control. Similar results were observed in a study by Xu et al. (44), where the microbial community of the control group formed a distinct cluster, suggesting that microbiota from the control differed from those of the probiotic treated group.

Microbial abundance slightly varied across treatments, with *Bacteroidetes* and *Firmicutes* being the most abundant phyla in all treatments. The results were similar to that of Peng et al. (45), where they observed *Bacteroidetes* (57%) being the most abundant phylum in the rumen, followed by *Firmicutes* (35%) and *Proteobacteria* (3.9%). Another study observed that *Bacteroidetes* were predominant in the rumen followed by *Firmicutes* and the findings suggested that a high concentrate diet provided a conducive environment for *Bacteroidetes* survival, though there was no significant difference between their treatments (33). The richness of phylum Lentisphaerae, Tenericutes, and Fibrobacteres was varied among the treatments, indicating that the treated groups affected the distribution of bacteria in the rumen.

Typically, *Proteobacteria* proportion is < 4% of the entire ruminal population, and an increase in the *Proteobacteria* population may cause subacute ruminal acidosis (46, 47). *Proteobacteria* was the most prevalent in T2, T3, and T5, while it was less abundant in T1 and T4. The low *Proteobacteria* abundance in the combination of the potential probiotic treated group indicates that the synergistic probiotics might be contributing to the balance of rumen microbial community at the phylum level. This is in accordance with results in a study by Pinloche et al. (48), as they observed a significant decrease in Proteobacteria occurrence in the highest level of yeast fed to the lactating cows. There is no clear indication of the cause of the decrease in *Proteobacteria* in the negative control. A closer look at both breeds in the negative control indicated that Damara accounted for about 14.5% of *Proteobacteria* before the trial, and at the end of 30 days, it only accounted for 2.8%, while Meatmaster had a slight decrease from 9.42–6.7%. This phylum consists of most pathogenic bacteria and the decrease might be because Damara sheep are known to be very adaptive to harsh conditions and resistant to parasites (49). On the contrary, in T2, T3, and T5, where *Proteobacteria* was most abundant, Damara sheep was the dominating breed as it resulted in the increase of this phylum after 30 days in these treatment groups, as compared to Meatmaster, which showed a decrease in all treatments. Even though the combination of potential probiotics treatment did not lead to any significant change of the core rumen microbial composition, the combination of potential probiotics (T4) was found to have stabilized the rumen microbiota and reduced the risk of pathogen colonization as compared to the singular probiotic groups and antibiotic group.

Genera *Prevotella, Clostridium*, CF231, BF311, *Fibrobacter, Methanobrevibacter, Succinispira*, and *Treponema* were found in all treatments and these genera belong to *Firmicutes, Bacteroidetes, Fibrobacteres, Proteobacteria, Spirochaetes*, and *Euryarchaeota. Prevotella* assist by utilizing feed proteins in the rumen and they are known to work in combination with cellulolytic species *Fibrobacters* in hemicellulose utilization (50, 51). Previous studies reported that *Treponema* is a genus of the primary bacterial community in the rumen and it is said that the genus break down plant polysaccharides from ingested feed (52). *Methanobrevibacter* produces CH4 by utilizing formate, CO_2_, H_2_ as substrates (53).

Antibiotic treated group (T5) and *L. rhamnosus* PT10 group (T3) exhibited aerobic bacterial genus *Acinetobacter*. Previous studies mostly found this genus in the small intestine mucosa, as it shows the presence of oxygen in the intestinal epithelial cell (54) and it represents a possible exclusion mechanism of strictly anaerobic and oxygen-sensitive microbes (55). The results suggest that the combination of potential probiotics was most effective in balancing the rumen microbial flora and improving the composition of the microbiota.

As revealed by the co-occurrence network, different rumen modules present a complex synergistic and unwieldy relationship. Three microbial taxa were observed to interact with other communities in their respective modules, suggesting that they may be keystone species of the rumen microbiota. Among the three keystone species observed, two (unclassified *Bacteroidetes* and *Clostridiales*) belong to the two most abundant phyla in rumen environment and had been widely studied through literature (2, 56, 57). However, the third keystone is from candidate phylum *SR1*; currently, there are no cultivated representatives available of rather extremely difficult to attain; only 16S rRNA gene information available (58). Nevertheless, this species occurrence is interesting as they appeared to present a significant shift in the network. Co-occurrence networks represent associations between microbial communities and help us understand the microbial ecology (59). The associations between microbial taxa may follow a sequence of universal dynamics as recently anticipated in the human microbiota (58).

The results from this study revealed that the administration of feed additives slightly altered rumen microbial community structure and abundance in the form of lactic acid as putative probiotics as well as increase in body weight gain. Although treatment groups were not diverse, their interactions showed that there were variations in the microbial communities. The synergism of probiotics demonstrated an improved microbial composition, which shows that probiotic administration can help maintain the balance of gut microbiota and enhance ruminal bacterial composition and structure (48). Breed variation affected rumen microbial composition and structure. This variation can be further investigated and explored to improve fitness traits in sheep production and breeding.

## Data Availability Statement

The datasets presented in this study can be found in online repositories. The names of the repository/repositories and accession number(s) can be found below: https://www.ncbi.nlm.nih.gov/, PRJNA578022.

## Ethics Statement

The animal study was reviewed and approved by Agricultural Research Council - Animal Production Institute Ethics committee (APIEC17/21). The trials were done at the Agricultural Research Council (ARC) GI Microbiology and Biotechnology unit and the Small Stocks Unit in Irene, Gauteng province.

## Author Contributions

SM conducted the study for her PhD research and drafted the manuscript. OA and MA designed, supervised, and proofread the manuscript. All authors contributed to the article and approved the submitted version.

## Conflict of Interest

The authors declare that the research was conducted in the absence of any commercial or financial relationships that could be construed as a potential conflict of interest.

## Supplementary Material

Supplementary Figure 1 Rarefaction curves of operational taxonomic units (OTUs at 97% sequence similarity), based on the 16S rRNA gene sequencing.
