# Supplementary figures and images for: Characterization of Rumen Microbiota of Two Sheep Breeds Supplemented With Direct-Fed Lactic Acid Bacteria
Source: Front Vet Sci. 2021 Jan 15;7:570074. doi: 10.3389/fvets.2020.570074 (PMC7843511; doi:10.3389/fvets.2020.570074)

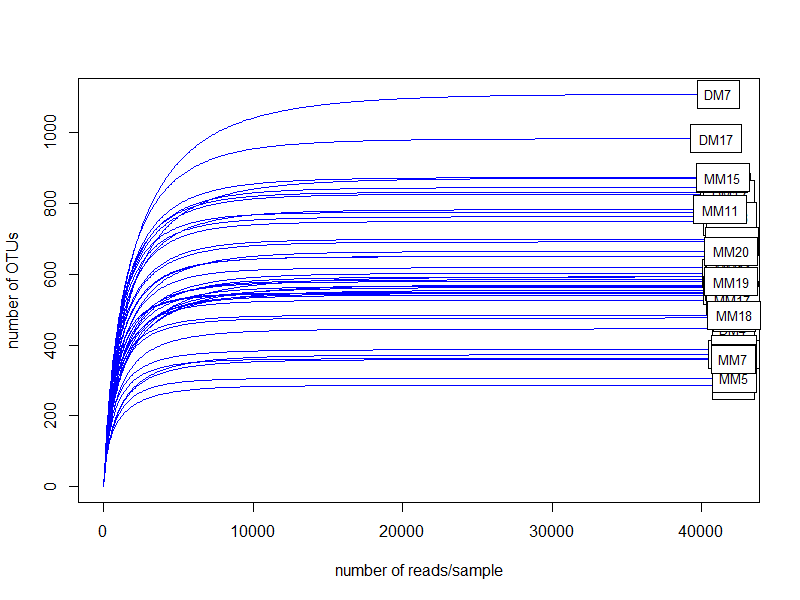

Supplement: Supplementary Figure 1 — Rarefaction curves of operational taxonomic units (OTUs at 97% sequence similarity), based on the 16S rRNA gene sequencing. [file Image_1.TIF]
